# Supplementary material for: Identification of common non-coding variants at 1p22 that are functional for non-syndromic orofacial clefting
Source: Nat Commun. 2017 Mar 13;8:14759. doi: 10.1038/ncomms14759 (PMC5355807; doi:10.1038/ncomms14759)
Supplement: Supplementary Information — Supplementary Figures and Supplementary Tables [file ncomms14759-s1.pdf]

Supplementary Information

Supplementary Figure 1

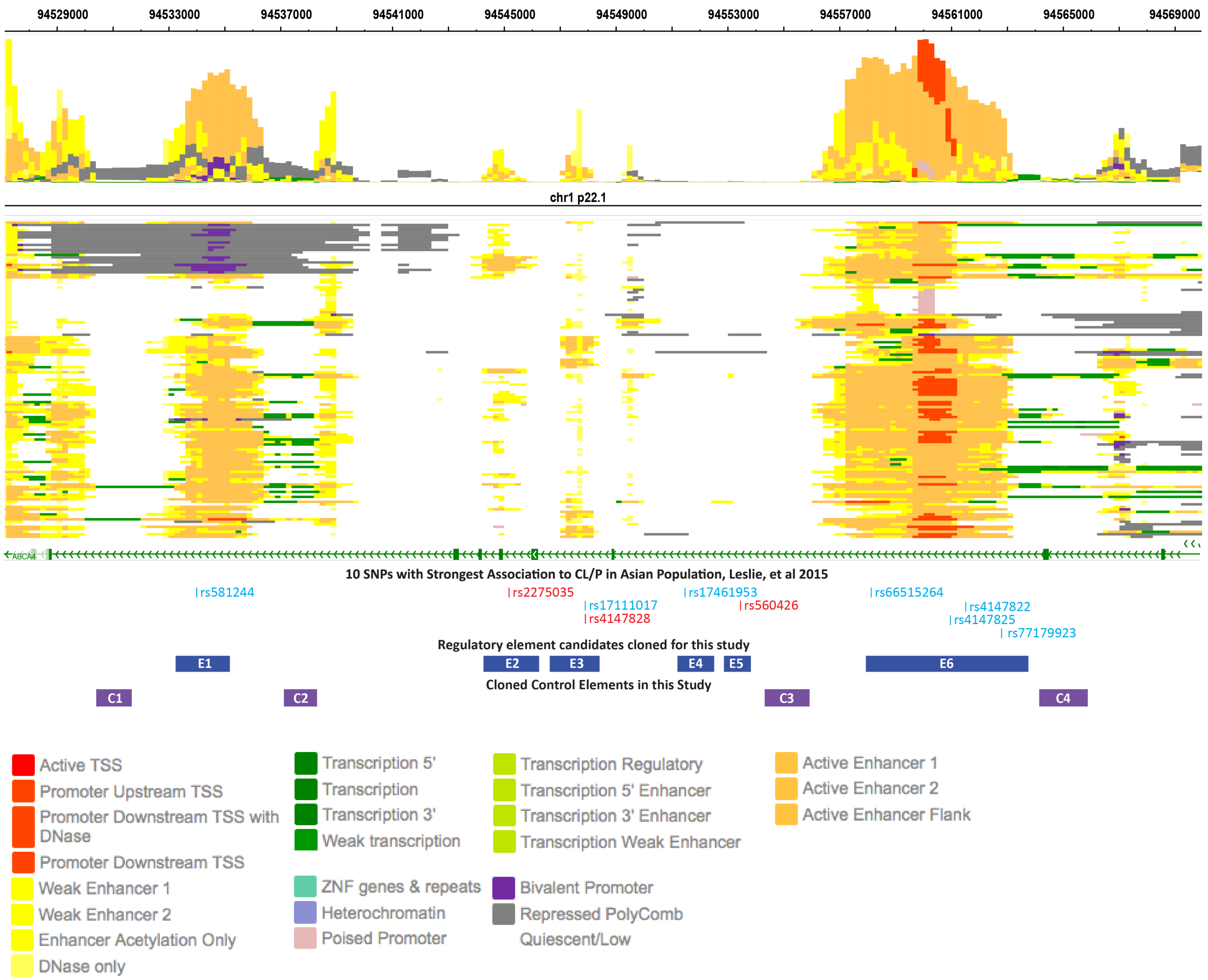

Supplementary Figure 1 Chromatin state annotations for the locus in **Fig. 1** across 127 reference epigenomes for cell and tissue types imputated by the Roadmap Epigenomics Project. Information on the colors used in the annotation was listed in the bottom.

Supplementary Figure 2

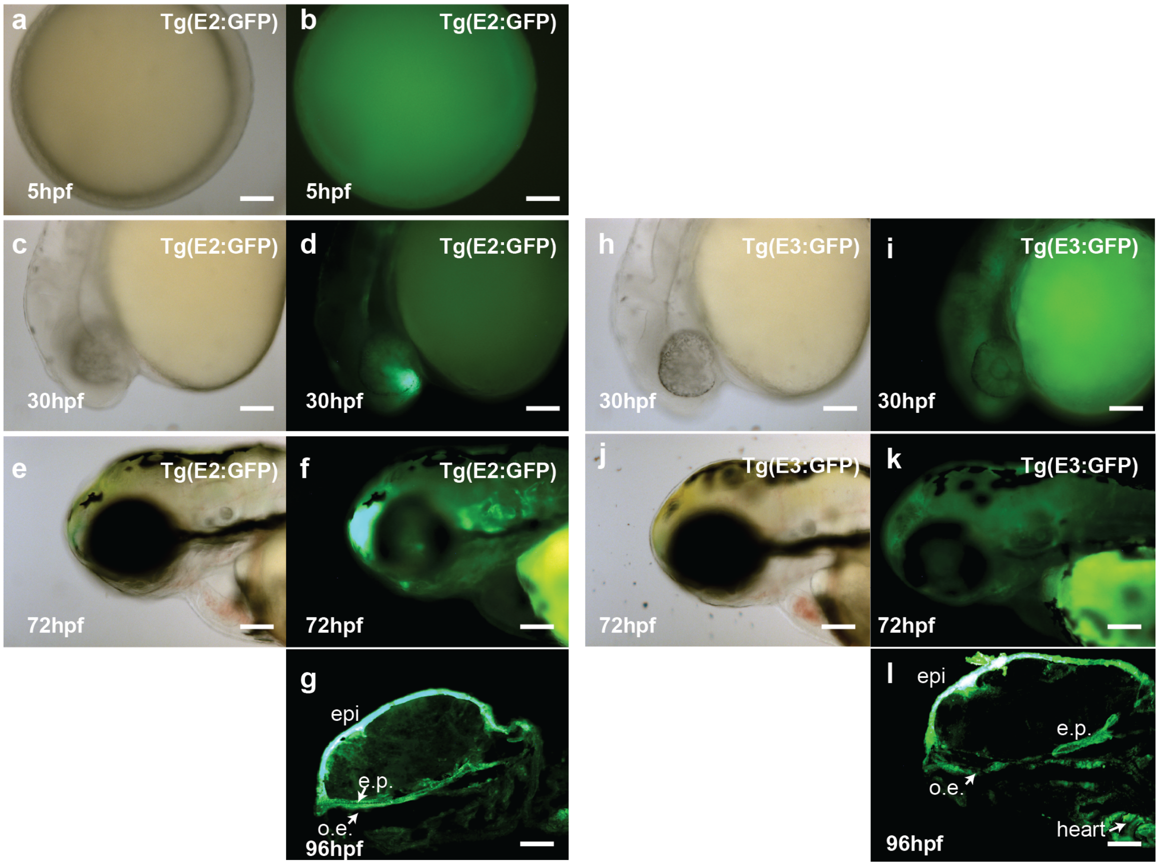

Supplementary Figure 2 Stable transgenic zebrafish lines showing in vivo enhancer activities patterns of E2 and E3 in different development stages. In 5hpf, F2 embryos of *Tg(E2:GFP)* exhibit universal enhancer activity (**a**, **b**). In 30 hpf, F2 embryos of *Tg(E2:GFP)* exhibit GFP activity in the forebrain (**c**, **d**), and F2 embryos of *Tg(E3:GFP)* exhibit GFP activity in central nervous system (**h**, **i**). In 72 hpf, F2 embryos of *Tg(E2:GFP)* exhibit GFP activity in lower jaw, otic vesicle, head epidermis and forebrain (**e**, **f**), and F2 embryos of *Tg(E3:GFP)* exhibit GFP activity in lower jaw and head epidermis (**j**, **k**). In 96hpf, sagittal section of F2 embryos of *Tg(E2:GFP*) (**g**) showing in vivo activity of E2 in oral epithelium (o.e.), ethmoid plate (e.p.) and head epidermis (epi), and sagittal section of F2 embryos of Tg(E3:GFP) (**l)** showing in vivo activity of E3 in oral epithelium, ethmoid plate, head epidermis and heart. Scale bar =100μm.

Supplementary Figure 3

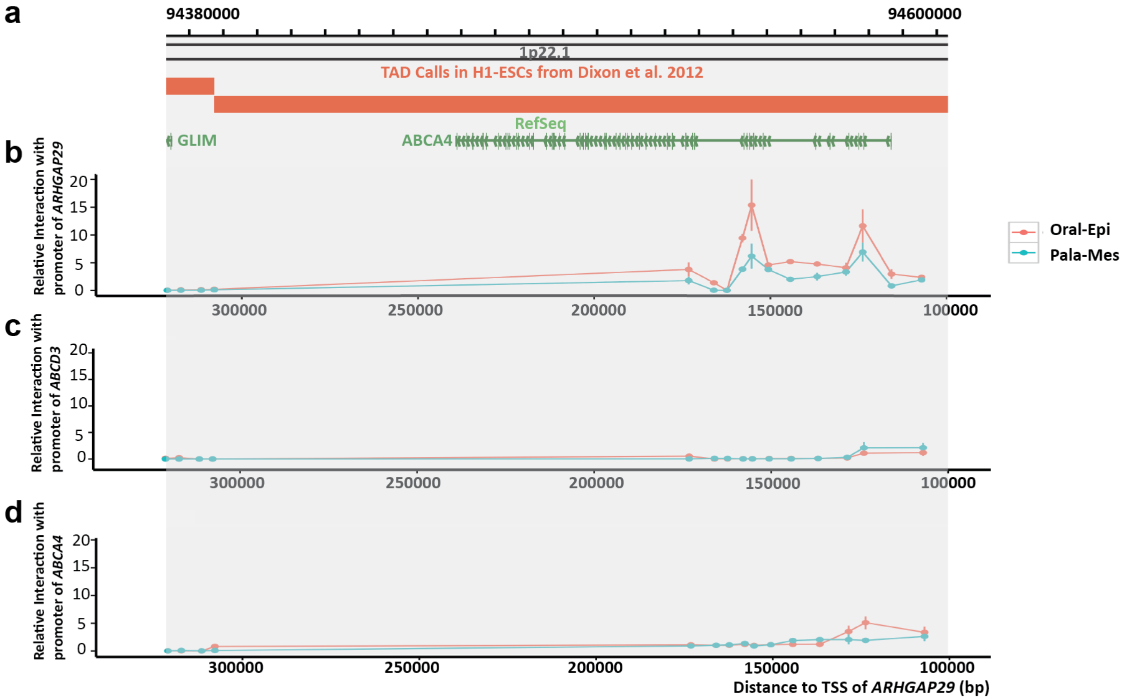

Supplementary Figure 3 Chromatin interactions at the 1p22 NSCL/P-associated region with *ARHGAP29* but not the region in the adjacent TAD. **a**, TAD covering all the EcoRI digested fragments investigated in this figure (drawn to scale). Interaction of the promoter of *ARHGAP29* (**b**), *ABCD3* (**c**), *ABCA4* (**d**) with EcoRI digestion fragments in 1p22 NSCLP-associated region and the neighboring TAD.

Supplementary Figure 4

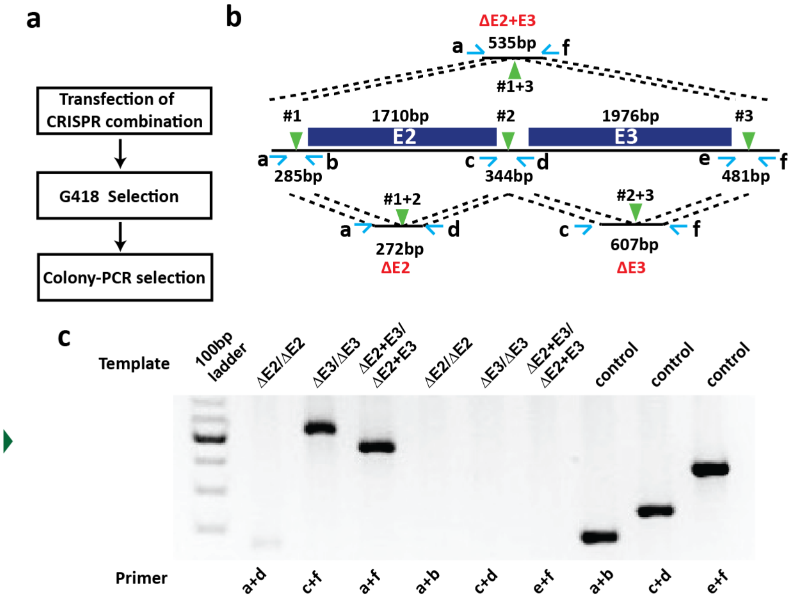

Supplementary Figure 4. Strategy on CRISPR-Cas9-mediated enhancer knockout and colony selection. a, general workflow of in vitro enhancer knockout. **b**, primer design for colony-PCR selection, figure is not drawn to scale. #1, #2, and #3 represent 3 gRNAs used for targeting enhancer E2, and E3. When co-transfecting gRNA #1 and #2 (#1+2), enhancer E2 will be deleted (ΔE2), and #1+3 will generate ΔE2+E3, #2+3 will generae ΔE3. In wildtype colonies (control in **c**), colony-PCR using primer a+b will generate 285bp product, c+d will generate 344bp product, primer e+f will generate 481bp product. In homozygous E2-deleted (ΔE2/ΔE2) colonies, primer a+b will not have any product (since binding site of primer b was deleted), a+d will generate 272bp product. In homozygous E3-deleted (ΔE3/ΔE3) colonies, primer c+f will generate 607bp product, while c+d will generate any product. In homozygous E2-and-E3-deleted (ΔE2+E3/ΔE2+E3) colonies, primer a+f will generate 535bp product.

Supplementary Figure 5

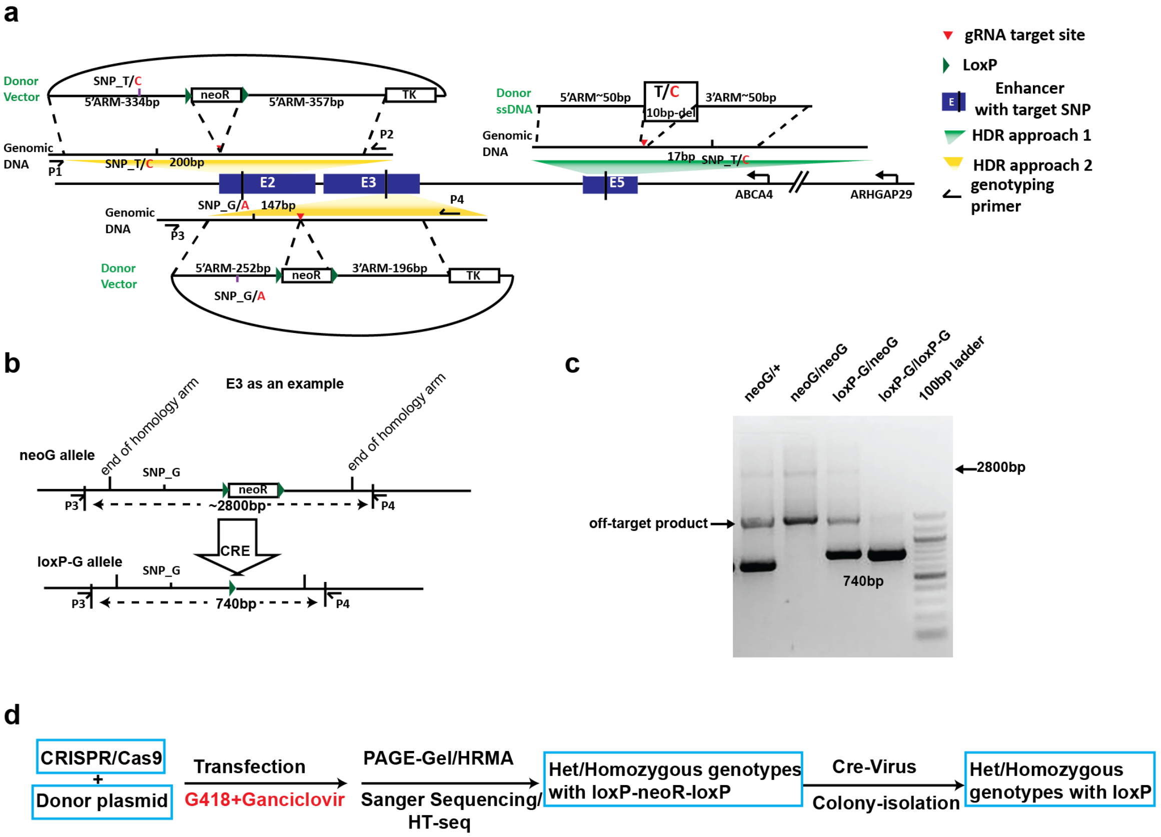

Supplementary Figure 5. Strategy on CRISPR-Cas9-mediated knock-in and colony selection. **a**, general overview of two approaches used in this study for CRISPR-Cas9-mediated knock-in. **b** and **c**, E3 as an example showing primer design for colony-PCR selection. d, strategy for colony screening of approach 2.

Supplementary Figure 6

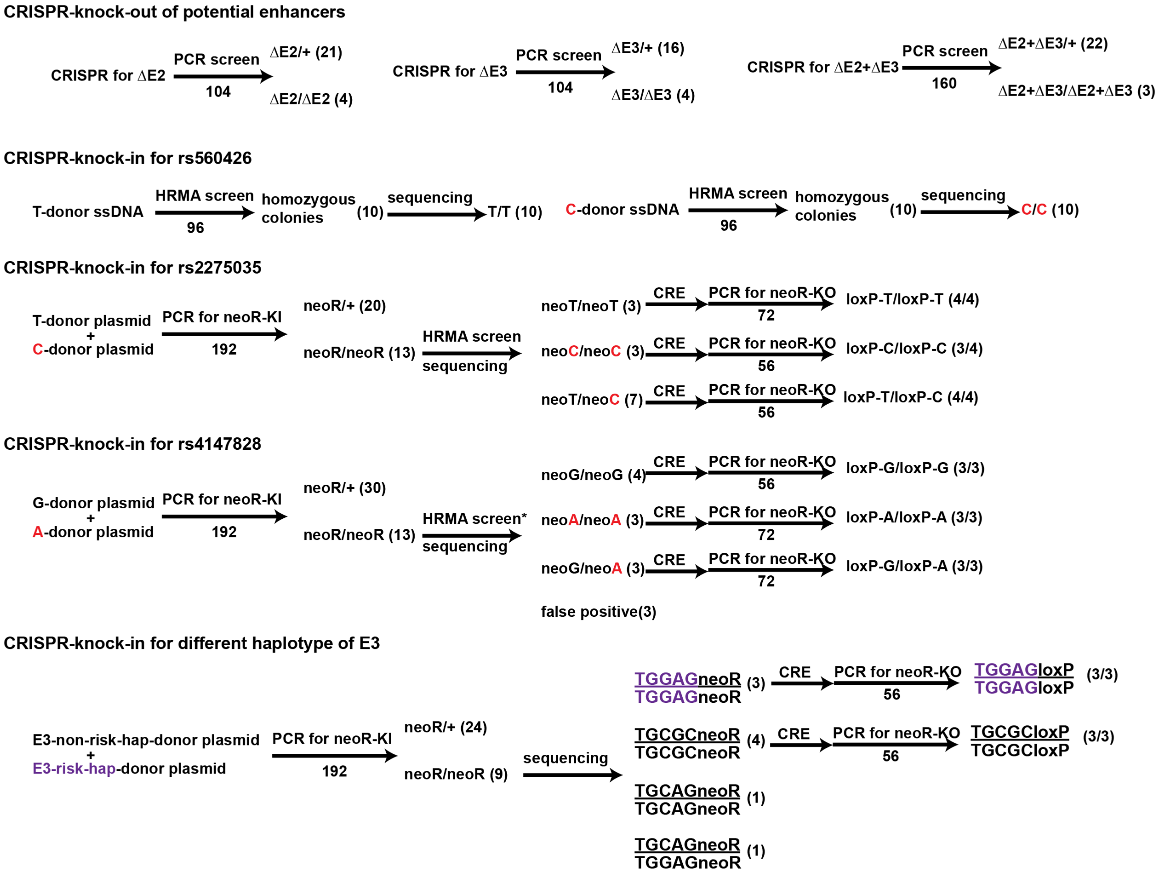

Supplementary Figure 6. Number of cell colonies screened and number of positive colonies after each selection. Number below each arrow indicate the total number of colonies screened using the method list above each arrow. Numbers of the colonies with the indicated genotype were listed besides the genotype. Red color indicates risk allele of each SNP, and purple color indicate the risk haplotype.

Supplementary Figure 7

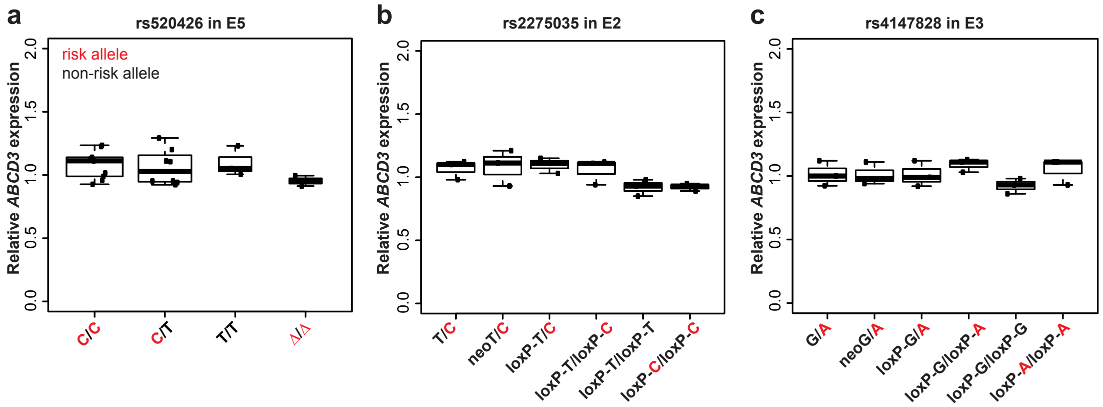

Supplementary Figure 7. Relative *ABCD3* expression in GMSM-K cells with different genotypes. Each dot represents mean expression of *ABCD3* in one isolated colony.

Supplementary Figure 8

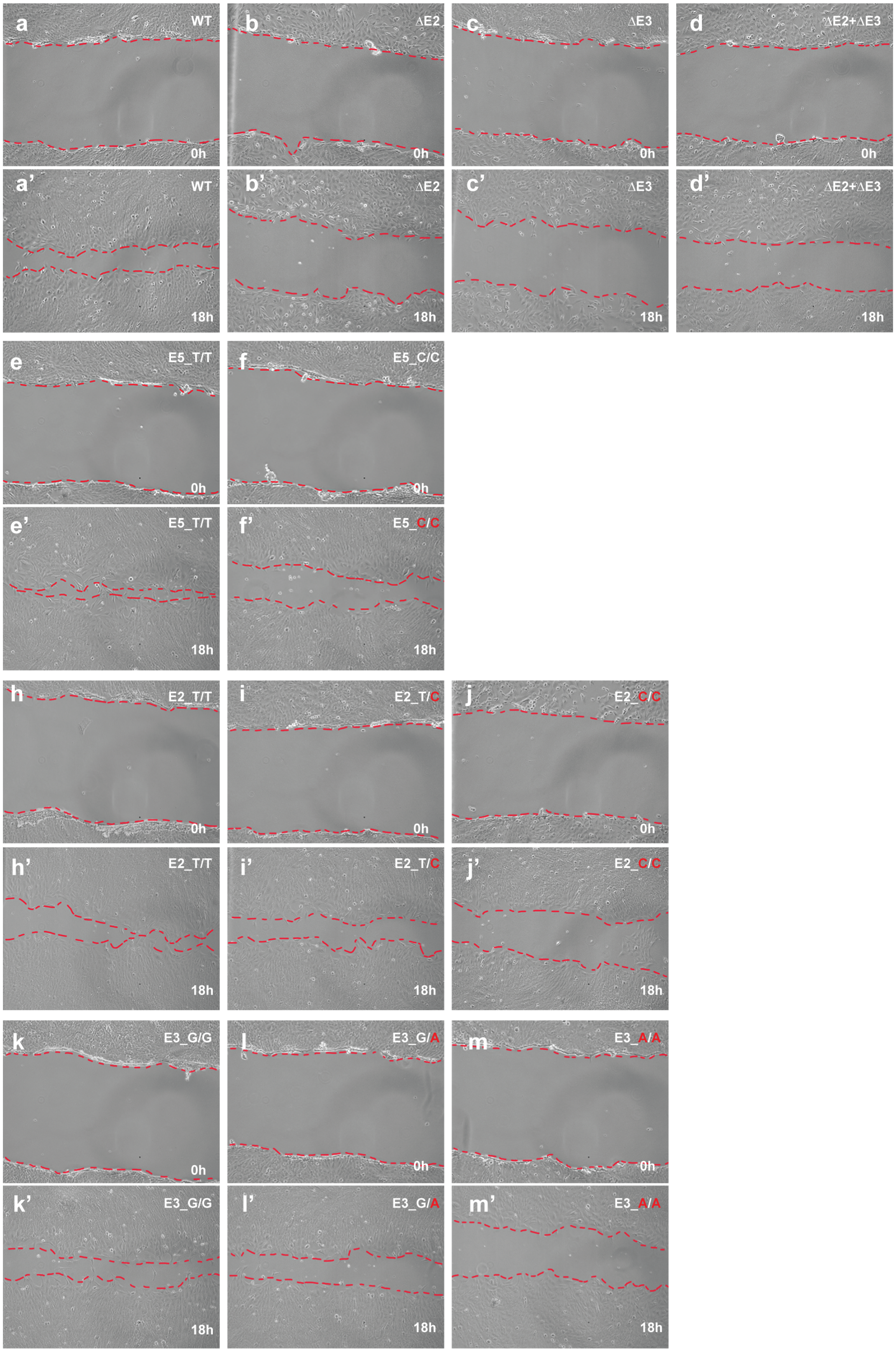

Supplementary Figure 8. Wound healing assay of oral epithelium cells with different genotype.

Supplementary Figure 9

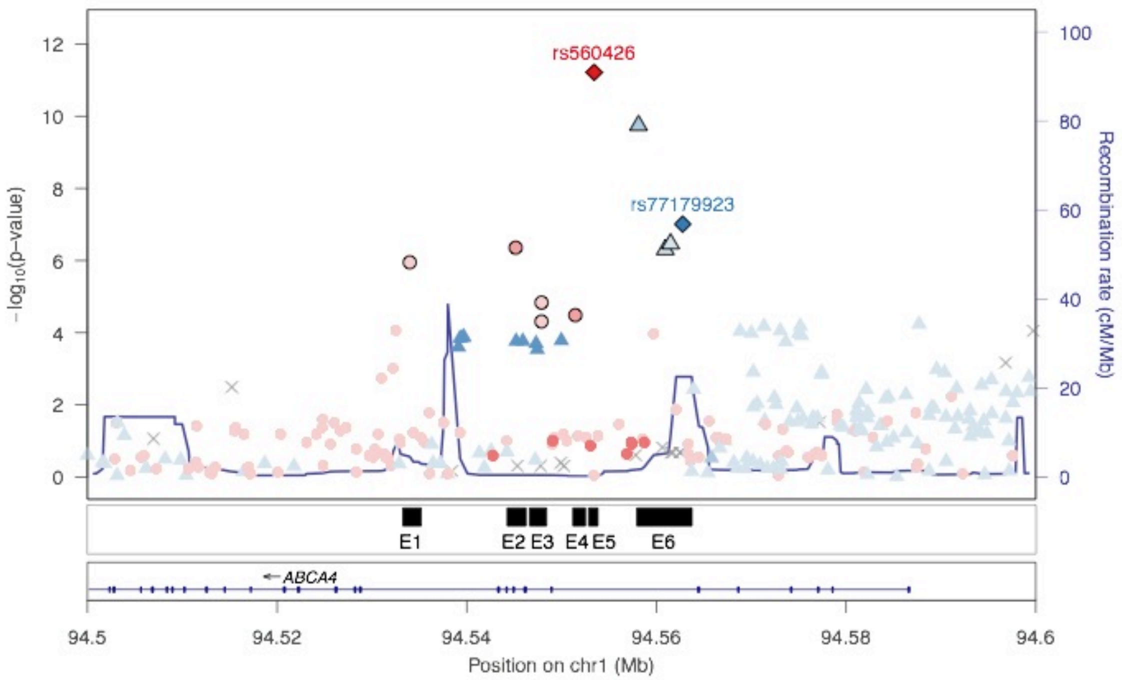

Supplementary Figure 9. Functional study pipeline proposed in this study. Experiments circled in green were illustrated in this study following the GWAS and fine mappings.

Supplementary Figure 10

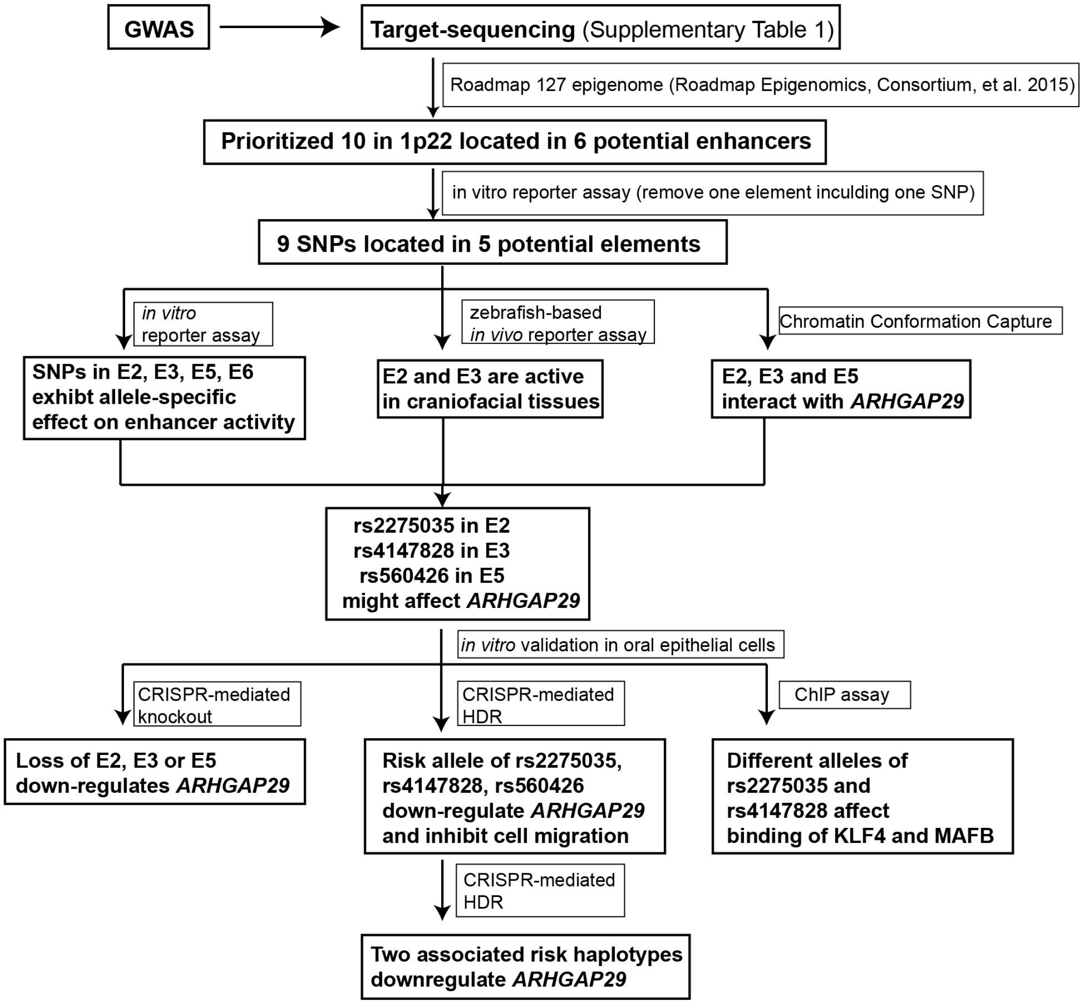

Supplementary Figure 10. Functional study pipeline proposed in this study.

Supplementary Table 1. Top Ten Most Significant Common SNPs in 1p22 Among Asian Trios

Enhancer Candidate	SNP	BP	Risk Allele	Non-Risk Allele	LD with rs560426		Risk Allele Frequency	P-value *TDT in Asian Trios*
					r^2	D'		
E1	rs581244	94533990	A	T	0.46	0.80	37.5%	1.12E-06
E2	rs2275035	94545160	C	T	0.62	0.98	56.6%	4.40E-07
E3	rs17111017	94547883	G	C	0.21	0.99	15.3%	4.90E-05
	rs4147828	94547889	A	G	0.41	0.97	65.9%	1.46E-05
E4	rs17461953	94551450	C	A	0.24	0.99	16.7%	3.27E-05
E5	rs560426	94553438	C	T	-	-	45.9%	6.06E-12
E6	rs66515264	94558110	T	G	0.47	0.99	28.5%	1.76E-10
	rs4147825	94560938	G	A	0.27	0.59	39.8%	4.87E-07
	rs4147822	94561489	A	G	0.27	0.60	38.7%	3.36E-07
	rs77179923	94562770	T	C	0.09	0.80	10.6%	9.79E-08

BP: base pair in chr1

Supplementary Table 2. Summary of function analysis for Top 10 associated NS CL/P Common SNPs in 1p22

Candidate	Luciferase assay in O.E.	Luciferase assay in P.M.	Craniofacial enhancer activity	Effect of risk allele on ARHGAP29	Effect of risk allele on cell migration
E1	+++*	++	NA		
rs581244	no changes	no changes			
E2	++	+	Yes		
rs2275037	risk allele decrease activity	no changes	No significant difference in F0	Decrease	inhibition
E3	++	+	Yes		
rs17111017	no changes	no changes			
rs4147828	risk allele decrease activity	no changes	No significant difference in F0	Decrease	inhibition
E4	-	-	NA		
rs17461953	not studied	not studied			
E5	++	+	NA		
rs560426	risk allele decrease activity	risk allele decrease activity		Decrease	inhibition
E6	++	+	NA		
rs66515264	no changes	risk allele increase acitivity			
rs4147825	no changes	no changes			
rs4147822	no changes	no changes			
rs77179923	no changes	no changes			

*+++: relative luciferase activity higher than 10

++: relative luciferase activity higher than 5 but less than 10

+: relative luciferase activity higher than baseline but less than 5

-: relative luciferase activity not significantly different with baseline level

Supplementary Table 3. Zebrafish-based in vivo assay for all potential enhancers

Element	GFP pattern at 4dpf	Total injected
E1	Notochord(62) Skin (50)	262
E2	Skin(42), Notochord (32), blood vessel(42), pharygeal arch (52), brain (32)	320
E3	Skin(33), Heart(42), blood vessel(52), retina(21), muscle (31), pharyngeal arch(42), brain (42)	332
E4	NA	242
E5	NA	262
E6	Neuron(64)	260

Note: neuron pattern could be the background activity of minimal promoter
NA: not detectable

Supplementary Table 4. Zebrafish-based in vivo assay for E2 with different alleles

Element	Replicate	Total injected	Pattern				
			Skin	Notochord	Blood Vessel	Pharygeal Arch	Brain
E2-non-risk	replicate_1	142	33	34	33	21	21
	replicate_2	141	31	31	32	18	22
	replicate_3	138	36	33	32	17	17
E2-risk	replicate_1	132	24	31	21	18	22
	replicate_2	136	31	33	33	14	23
	replicate_3	142	22	29	28	16	15

Supplementary Table 5. Zebrafish-based in vivo assay for E3 with different alleles

Element	Replicate	Total injected	Pattern							
			skin	Heart	Blood Vessel	Retina	Muscle	gut	pharyngeal arch	brain
E3-non-risk	replicate_1	125	23	42	42	24	15	32	23	22
	replicate_2	132	25	44	32	33	14	21	22	32
	replicate_3	152	33	52	25	21	19	22	28	35
E3-risk	replicate_1	130	21	52	41	24	17	22	21	25
	replicate_2	122	21	44	33	21	21	20	20	22
	replicate_3	132	22	45	32	22	22	19	22	20

Supplementary Table 6. Haplotype Association Analysis of 4 functional SNPs in 633 Filipino trios

Haplotype	# Transmitted	# Untransmitted	χ^2	P-value	SNP1	SNP2	SNP3	SNP4
CACG	220	204	0.6	0.4371	rs2275035	rs4147828	rs560426	rs66515264
CACT	423	298	21.67	**3.24E-06**	rs2275035	rs4147828	rs560426	rs66515264
CATG	130	150	1.43	0.2320	rs2275035	rs4147828	rs560426	rs66515264
CATT	0	1	1	0.3173	rs2275035	rs4147828	rs560426	rs66515264
CGCG	4	0	4	0.0455	rs2275035	rs4147828	rs560426	rs66515264
CGTG	0	3	3	0.0833	rs2275035	rs4147828	rs560426	rs66515264
TATG	109	133	2.38	0.1229	rs2275035	rs4147828	rs560426	rs66515264
TATT	1	0	1	0.3173	rs2275035	rs4147828	rs560426	rs66515264
TGCG	5	7	0.33	0.5637	rs2275035	rs4147828	rs560426	rs66515264
TGTG	373	470	11.16	**8.35E-04**	rs2275035	rs4147828	rs560426	rs66515264
TGTT	1	0	1	0.3173	rs2275035	rs4147828	rs560426	rs66515264

*red denotes risk allele

Supplementary Table 7. Haplotypes for all common SNPs in E3

	rs75017380	rs3789419	rs17111017	rs4147828	rs4147827	T	U	P
H1	C	A	C	A	C	174	128	0.0081
H2	T	G	C	A	C	460	482	0.51
H3	T	G	C	G	C	380	479	0.0007
H4	T	G	G	A	G	228	158	0.0004

Supplementary Table 8. BACs used in this study

BAC end ID	Usage
RP11-979G24	test 3C primer anchoring to the promoter of ARHGAP29
RP11-109C4	test 3C primer anchoring to the promoter of ABCA4
RP11-937N17	test 3C primer anchoring to the promoter of ABCD3 and primer anchoring near the potential enhancers
RP11-826E4	test 3C primer anchoring near GLIM

Supplememtary Table 9. Primer for Cloning and site-diret mutagenesis of the potential enhancers

Element	Forward(5'-3')		Usage
C1	GCCACAGAAGCTAATGTAGG	CACCACCCTCAACAGACCCAAACTA	Clone of full-length of C1
C2	TAGCAGCTTCAATATCAGTC	CACCAGCAAAAGGTTAGGCCTGGCA	Clone of full-length of C2
C3	TCAAAGACTGGAATCCAAAGA	CACCGCCCTTGAGTTTCATTCAACA	Clone of full-length of C3
C4	CCACATACCTAATGGTGAACC	CACCACTAGCTGCTTTTAGTGGCT	Clone of full-length of C4
	TCAGTGGCATTGCTGACTGC	CACCTCTACTGGACATCATTTCC	Clone of full-length of E1
E1	GGGAAGCCACCTGCCATGCAGCAAGTGACCT	AGGTCACTTGCTGCATGGCAGGTGGCTTCCC	site-direct mutagenesis for rs581244(T>A)
	GCCTAAAACCAGACAGAGATC	CACCGACTTGCTGTGGGTGAC	Clone of full-length of E2
E2	ATAGGCTGTACCTCACCAATAACAA	TTGTTATTGGTGAGGTACAGCCTAT	site-direct mutagenesis for rs2275035(C>T); note this pair of primers were used to mutate the risk allele to non-risk allele, since the original genotype of rs2275035 is C(risk allele)
	AAATTAGCCGGACATGGTGG	CACCATCTGATTGCACAAGTCTCC	Clone of full-length of E3
	TCACAAATGATTAGACTTGCTATCTAGACAG	CTGTCTAGATAGCAAGTCTAATCATTTGTGA	site-direct mutagenesis for rs17111017(G>C); note this pair of primers were used to mutate the risk allele to non-risk allele, since the original genotype of rs17111017 is G(risk allele)
E3	ATGATTAGACTTGCTGTCTAGACAGTCAAGA	TCTTGACTGTCTAGACAGCAAGTCTAATCAT	site-direct mutagenesis for rs4147828(A>G); note this pair of primers were used to mutate the risk allele to non-risk allele, since the original genotype of rs4147828 is A(risk allele)
E4	TCCATGGCTCCCTACTGTCTC	CTGCCAGATCTGTGGGAAATA	Clone of full-length of E4
	ACACACCAACCCATTCCATT	CACCGGCAAAAGCAGTGTCACAGA	Clone of full-length of E5
E5	TGGGGTTTACCTGCAAGAGTCTCCC	GGGAGACTCTTGCAGGTAAACCCCA	site-direct mutagenesis for rs560426(T>C)
	GCAACTGGGAGAATGTGGAT	CACCGCATCCCAGACAGACTGCTT	Clone of full-length of E6
	AATCACTGGGGGGTTTGGGGACATGTCAAAC	GTTTGACATGTCCCCAAACCCCCCAGTGATT	Site-direct mutagenesis for rs66515264(G>T)
	AGATGCCGTCACGGTGGTTTACTGA	TCAGTAAACCACCGTGACGGCATCT	Site-direct mutagenesis for rs4147825(G>A)
	GGTTAACTGAGGCCGGCTACGGCAGAGCTTT	AAAGCTCTGCCGTAGCCGGCCTCAGTTAACC	Site-direct mutagenesis for rs4147822(A>G); note this pair were used to mutate the risk allele to non-risk allele, since the original genotype of rs4147822 is A(risk allele)
	GGTGGCATCCCTAGGTTCTGACCCTTCATAC	GTATGAAGGGTCAGAACCTAGGGATGCCACC	Site-direct mutagenesis for rs77179923(C>T)
	AAGAGGCTGTGCTTTCATAAGAGCAAAATGC	GCATTTTGCTCTTATGAAAGCACAGCCTCTT	Site-direct mutagenesis for rs952499(T>C). In order to make E6 with non-risk haplotype
E6	TCCCAGACACTTGACATAGCAATATCCACCC	GGGTGGATATTGCTATGTCAAGTGTCTGGGA	Site-direct mutagenesis for rs2068334(G>A). In order to make E6 with non-risk haplotype.

Supplementary Table 10. Primer for 3C-qPCR

coordinate of EcoRI site(hg19)	Detection of EcoRI fragment interacting ARHGAP29 (NM_001328667.1) promoter_1	
	Forward(5'-3')	Reverse(5'-3')
chr1:94381251-94381256	TGCGATTATAGGCACCCATT	ATCTGTAGGTCGCCAGCATC
chr1:94383192-94383197	TGCGATTATAGGCACCCATT	AGTTGCTGCGAAGAGCAAA
chr1:94388854-94388859	TGCGATTATAGGCACCCATT	TGGCTGTTCAACCCTACTCA
chr1:94394704-94394709	TGCGATTATAGGCACCCATT	AGTGGTGGCTTGGTAGTGGT
chr1:94523439-94523444	TGCGATTATAGGCACCCATT	TCCAGGGAACTACTGCCAAC
chr1:94536847-94536852	TGCGATTATAGGCACCCATT	TGATTGGATTTTCCTGCTTG
chr1:94539232-94539237	TGCGATTATAGGCACCCATT	GCAGCTTGTTCCTAATGCAG
chr1:94543943-94543948	TGCGATTATAGGCACCCATT	AGTGGGGTCACTGACTTTGG
chr1:94547106-94547111	TGCGATTATAGGCACCCATT	CACGAGAAAACCACAAACCA
chr1:94548914-94548919	TGCGATTATAGGCACCCATT	TCTGAGATCTTGGGGAGGAA
chr1:94558084-94558089	TGCGATTATAGGCACCCATT	TTCCCTCTCATTCTGGGAGA
chr1:94559853-94559858	TGCGATTATAGGCACCCATT	GCCCATCAATGTCCTGTTTC
chr1:94573349-94573354	TGCGATTATAGGCACCCATT	TGGCTGTGGTCAGAAAGTGA
chr1:94576223-94576228	TGCGATTATAGGCACCCATT	GTCGAATCCCACAGGAAGAG
chr1:94592569-94592574	TAGAGGCAGGGTTTCGCTAT	TCTGTGCCAAATGGTTTTGA
coordinate of EcoRI site(hg19)	Detection of EcoRI fragment interacting ARHGAP29 (NM_001328667.1) promoter_2	
	Forward(5'-3')	Reverse(5'-3')
chr1:94381251-94381256	ATCTGTAGGTCGCCAGCATC	AGTCGTGGGGGATAGGAAGT
chr1:94383192-94383197	GCTGCGAAGAGCAAATGAGT	AGTCGTGGGGGATAGGAAGT
chr1:94388854-94388859	TGGCTGTTCAACCCTACTCA	AGTCGTGGGGGATAGGAAGT
chr1:94394704-94394709	TCTCGTAGTGGTGGCTTGGT	AGTCGTGGGGGATAGGAAGT
chr1:94523439-94523444	TCCAGGGAACTACTGCCAAC	AGTCGTGGGGGATAGGAAGT
chr1:94536847-94536852	TGATTGGATTTTCCTGCTTG	AGTCGTGGGGGATAGGAAGT
chr1:94539232-94539237	GCAGCTTGTTCCTAATGCAG	AGTCGTGGGGGATAGGAAGT
chr1:94543943-94543948	AGTGGGGTCACTGACTTTGG	AGTCGTGGGGGATAGGAAGT
chr1:94547106-94547111	CACGAGAAAACCACAAACCA	AGTCGTGGGGGATAGGAAGT
chr1:94548914-94548919	TCTGAGATCTTGGGGAGGAA	AGTCGTGGGGGATAGGAAGT
chr1:94558084-94558089	TTCCCTCTCATTCTGGGAGA	TCGTGGGGGATAGGAAGTTT
chr1:94559853-94559858	GCCCATCAATGTCCTGTTTC	AGTCGTGGGGGATAGGAAGT
chr1:94573349-94573354	TGGCTGTGGTCAGAAAGTGA	AGTCGTGGGGGATAGGAAGT
chr1:94576223-94576228	GTCGAATCCCACAGGAAGAG	AGTCGTGGGGGATAGGAAGT
chr1:94592569-94592574	TCCACTTTGAGGCGCTTTAG	AGTCGTGGGGGATAGGAAGT

coordinate of EcoRI site(hg19)	Detection of EcoRI fragment interacting ABCA4((NM_000350) promoter	
coordinate of EcoRI site(hg19)	Forward(5'-3')	Reverse(5'-3')
chr1:94381251-94381256	TGGCCAATGAATCAAGTGAA	ATCTGTAGGTCGCCAGCATC
chr1:94383192-94383197	TGGCCAATGAATCAAGTGAA	GCTGCGAAGAGCAAATGAGT
chr1:94388854-94388859	TGGCCAATGAATCAAGTGAA	TGGCTGTTCAACCCTACTCA
chr1:94394704-94394709	TGGCCAATGAATCAAGTGAA	TCTCGTAGTGGTGGCTTGGT
chr1:94523439-94523444	TGGCCAATGAATCAAGTGAA	TCCAGGGAACTACTGCCAAC
chr1:94536847-94536852	TGGCCAATGAATCAAGTGAA	TGATTGGATTTTCCTGCTTG
chr1:94539232-94539237	TGGCCAATGAATCAAGTGAA	GCAGCTTGTTCCTAATGCAG
chr1:94543943-94543948	TGGCCAATGAATCAAGTGAA	AGTGGGGTCACTGACTTTGG
chr1:94547106-94547111	TGGCCAATGAATCAAGTGAA	CACGAGAAAACCACAAACCA
chr1:94548914-94548919	TGGCCAATGAATCAAGTGAA	TCTGAGATCTTGGGGAGGAA
chr1:94558084-94558089	TGGCCAATGAATCAAGTGAA	TCCCTCTCATTCTGGGAGAC
chr1:94559853-94559858	TGGCCAATGAATCAAGTGAA	GCCCATCAATGTCCTGTTTC
chr1:94573349-94573354	TGGCTGTGGTCAGAAAGTGA	TGGCTGTGGTCAGAAAGTGA
chr1:94576223-94576228	TGGCCAATGAATCAAGTGAA	GTCGAATCCCACAGGAAGAG
chr1:94592569-94592574	TGGCCAATGAATCAAGTGAA	GTTCCACTTTGAGGCGCTTT
coordinate of EcoRI site(hg19)	Detection of EcoRI fragment interacting ABCD3(NM_002858) promoter_1	
coordinate of EcoRI site(hg19)	Forward(5'-3')	Reverse(5'-3')
chr1:94381251-94381256	CCGTGGGACTTCAATGTCAG	ATCTGTAGGTCGCCAGCATC
chr1:94383192-94383197	CGTGGGACTTCAATGTCAGG	GCTGCGAAGAGCAAATGAGT
chr1:94388854-94388859	CGTGGGACTTCAATGTCAGG	TGGCTGTTCAACCCTACTCA
chr1:94394704-94394709	CGTGGGACTTCAATGTCAGG	TCTCGTAGTGGTGGCTTGGT
chr1:94523439-94523444	CGTGGGACTTCAATGTCAGG	TCCAGGGAACTACTGCCAAC
chr1:94536847-94536852	CCGTGGGACTTCAATGTCAG	TGATTGGATTTTCCTGCTTG
chr1:94539232-94539237	CCGTGGGACTTCAATGTCAG	GCAGCTTGTTCCTAATGCAG
chr1:94543943-94543948	ACTGCCGTGGGACTTCAAT	AGTGGGGTCACTGACTTTGG
chr1:94547106-94547111	CCGTGGGACTTCAATGTCAG	CACGAGAAAACCACAAACCA
chr1:94548914-94548919	CGTGGGACTTCAATGTCAGG	TCTGAGATCTTGGGGAGGAA
chr1:94558084-94558089	CCGTGGGACTTCAATGTCAG	TTCCCTCTCATTCTGGGAGA
chr1:94559853-94559858	CGTGGGACTTCAATGTCAGG	GCCCATCAATGTCCTGTTTC
chr1:94573349-94573354	CGTGGGACTTCAATGTCAGG	TGGCTGTGGTCAGAAAGTGA
chr1:94576223-94576228	CGTGGGACTTCAATGTCAGG	GTCGAATCCCACAGGAAGAG
chr1:94592569-94592574	CGTGGGACTTCAATGTCAGG	GTTCCACTTTGAGGCGCTTT
coordinate of EcoRI site(hg19)	Detection of EcoRI fragment interacting ABCD3 (NM_002858) promoter_2	

	Forward(5'-3')	Reverse(5'-3')
chr1:94381251-94381256	ATCTGTAGGTCGCCAGCATC	GTGCTGTGCCCTAGGTGTTT
chr1:94383192-94383197	GCTGCGAAGAGCAAATGAGT	GTGCTGTGCCCTAGGTGTTT
chr1:94388854-94388859	TGGCTGTTCAACCCTACTCA	GTGCTGTGCCCTAGGTGTTT
chr1:94394704-94394709	TCTCGTAGTGGTGGCTTGGT	GTGCTGTGCCCTAGGTGTTT
chr1:94523439-94523444	ATGTTCCTGGGAAGAGCACA	GCTGTGCCCTAGGTGTTTTC
chr1:94536847-94536852	TGATTGGATTTTCCTGCTTG	GTGCTGTGCCCTAGGTGTTT
chr1:94539232-94539237	GCAGCTTGTTCCTAATGCAG	GTGCTGTGCCCTAGGTGTTT
chr1:94543943-94543948	AGTGGGGTCACTGACTTTGG	GTGCTGTGCCCTAGGTGTTT
chr1:94547106-94547111	CACGAGAAAACCACAAACCA	GTGCTGTGCCCTAGGTGTTT
chr1:94548914-94548919	TCTGAGATCTTGGGGAGGAA	GTGCTGTGCCCTAGGTGTTT
chr1:94558084-94558089	CCTGGTAGAGACCGTTTGACA	GTGCTGTGCCCTAGGTGTTT
chr1:94559853-94559858	GCCCATCAATGTCCTGTTTC	GTGCTGTGCCCTAGGTGTTT
chr1:94573349-94573354	TGGCTGTGGTCAGAAAGTGA	GTGCTGTGCCCTAGGTGTTT
chr1:94576223-94576228	AGTCGAATCCCACAGGAAGA	GTGCTGTGCCCTAGGTGTTT
chr1:94592569-94592574	TCCACTTTGAGGCGCTTTAG	GTGCTGTGCCCTAGGTGTTT

Supplementary Table 11. qRT-PCR primer

Target genes	Forward(5'-3')	Reverse(5'-3')
ARHGAP29	GCCTAACTGCCAAGACTACAA	GGGTTGAGTGGCATTGATAGA
ABCA4	TCCAAGCACCTCCAGTTTATC	CCCAGCACTCACGGAATAAT
ABCD3	CATTGGTCGTAGCAGGAAAGA	CCTTACTCGGAAGCACAGTTTA
GAPDH	TGCACCACCAACTGCTTAGC	GGCATGGACTGTGGTCATGAG

Supplementary Table 12. **Primer for ChIP-qPCR**

ChIP antibody	Forward(5'-3')	Reverse(5'-3')	Usage
MAFB	AGATGAAGGGGCTGTTCTCA	CAATCCCCAAAGTATATCAACACTC	DNA fragment covering rs4147828
	CTCATGGCTACTGGGGTGTT	GGATAGGAACCGGAGGTAGC	Off-target region chr20:38,034,907-38,034,910(hg19)
	AGGTACGGGAGGCAACTAGC	GCCATTAGCCGCATCTAGTC	Positive control targeting chr1:94,703,328-94,703,330 (hg19)
KLF4	ATAAACCACAGGGCAAGTCG	ACTCCATGCTGGAGGATTTG	DNA fragment covering rs2275035
	CTCATGGCTACTGGGGTGTT	GGATAGGAACCGGAGGTAGC	Off-target region chr20:38,034,907-38,034,910(hg19), same as off-target control for MAFB ChIP-qPCR
	CCAGCTCTGTCCCTGAGTTC	TCTCTAGGGCGGTTCAGTGT	Positive control targeting chr1:94,510,876-94,511,769(hg19)

Supplementary Table 13. Oligos used for CRISPR/Cas9-mediated knockout of E2 and E3

gRNA target site	oligo sequence for ligating to px330	
	Forward(5'-3')	Reverse(5'-3')
chr1:94079068-94079089(hg38) (#1)	CACCGGGATATAATTCAAGCAAT	AAACATTGCTTGAATTATATCCC
chr1:94080777-94080799(hg38) (#2)	CACCGGGGCCAATGCTCCAACGTT	AAACAACGTTGGAGCATTGGCCCC
chr1:94082744-94082766(hg38)(#3)	CACCGGATTTGGTCATGACCCTGT	AAACACAGGGTCATGACCAAATCC
primer in Supplementary Figure 4 (for genotypeing)		
a	CTTCCTGGGCTGACAGAATC	
b	CACTGGAATGCTTTGCTTCA	
c	TGGAGTCAATCCCCAGAAAG	
d	GCAGCTGGATTAAGGATTGC	
e	CTGCGAATAATGGAGGGAGA	
f	TAAGCAACTTGCCTGGTGTG	

Supplementary Table 14. Oligos used for CRISPR/Cas9-mediated knockin

**For rs560426**		
**gRNA target site**	**oligo sequence for ligating to px330**	
	**Forward(5'-3')**	**Reverse(5'-3')**
chr1:94,553,419-94,553,438 (hg19)	**CACC**GAGGTAAACCCCATGAAGGG	**AAAC**CCCTTCATGGGGTTTACCTC
**Primer for PAGE-based genotyping**	TTACTCACAGCCAGGTGCAG	GGCAAAAGCAGTGTCACAGA
**Primer for HRMA**	ACACGGCAGTGAATGAAACA	CTCCTGTGGGAGAATCTTGC
**oligos for 10bp-deletion (5'-3')**	TACACAGCATGACGACAGTGATGGAAGGGCTGGCGAAGCCTCAGGGAGACAACCCCATGAAGGGAGGACTTTGTTTCATTCACTGCCGTGTCCCCAGCAC	
**For rs4147828**		
**gRNA target site**	**oligo sequence for ligating to px330**	
	**Forward(5'-3')**	**Reverse(5'-3')**
chr1:94,545,344-94,545,363 (hg19)	**CACC**GGCCAAACAAGGCCCCCGCT	**AAAC**AGCGGGGGCCTTGTTTGGCC
**Primer for cloing the 5' homogy arm**	**GCCATCGAT**TTAGTTCACAAATGATTAGA	**GCCGGATCC**CGAGTATGCACACTGATGTG
**Primer for cloing the 3' homogy arm**	**GCGTCTAGA**GCCCAGATTGAGATGCGTTT	**GCCAAGCTT**GACCCTGTGGGAAAGAGAGA
**Primer for HRMA**	GAATGTACAAACAATCCCCAAA	TAAACTGGGCCTTCTGCTTT
**For rs2275035**		
**gRNA target site**	**oligo sequence for ligating to px330**	
	**Forward(5'-3')**	**Reverse(5'-3')**
chr1:94,548,099-94,548,118 (hg19)	**CACC**GTCAGTGTGCATACTCGGCCC	**AAAC**GGGCCGAGTATGCACACTGAC
**Primer for cloing the 5' homogy arm**	**GCCATCGAT**AGACAAGGGACAGATTTTAC	**GCCGGATCC**GGGGCCTTGTTTGGCCTCTC
**Primer for cloing the 3' homogy arm**	**GCGTCTAGA**CGCTTGAAATCTCAGCTTGA	**GCCAAGCTT**TTCCAGGTGAAGCTCAGGTC
**Primer for HRMA**	ATAAACCACAGGGCAAGTCG	ACTCCATGCTGGAGGATTTG
